# Normalization of CEA, CA15‐3, and CA27‐29 Tumor Markers Following Single Lung Transplant in a Patient With a History of Early‐Stage Breast Cancer and Idiopathic Pulmonary Fibrosis: A Case Report

**DOI:** 10.1002/cnr2.70530

**Published:** 2026-05-14

**Authors:** Yolcar Chamorro, Charles L. Vogel, Ana C. Sandoval‐Leon

**Affiliations:** ^1^ Department of Medical Oncology Miami Cancer Institute/Baptist Health South Florida Miami Florida USA; ^2^ Department of Medical Oncology Miami Cancer Institute/Baptist Health South Florida Plantation Branch Plantation Florida USA

**Keywords:** biomarkers, breast cancer, CEA, idiopathic pulmonary fibrosis, lung transplant, tumor markers

## Abstract

**Background:**

The use of serum tumor markers can provide additional information about a patient's response to treatment, but their use has been controversial in the management of breast cancer (BC) because of a lack of standardization. CEA, CA15‐3, and CA27‐29 are used to monitor disease activity in metastatic BC (MBC), but they have poor sensitivity and specificity in early‐stage BC. Some oncologists or lung transplant centers may test these tumor markers to detect potential occult existing malignancy in patients who are eligible for transplant. However, these tests may provide inaccurate results and cause unnecessary delays in performing the transplant.

**Case Report:**

Herein we present a case of a 52‐year‐old woman who had early‐stage BC, underwent cancer treatment, and later developed idiopathic pulmonary fibrosis (IPF) that required a lung transplant. Her previous oncologist tested her tumor markers, which were elevated. Multiple scans over the years did not show any evidence of recurrence. After her single lung transplant, her serum tumor markers normalized.

**Discussion/Conclusion:**

Elevated tumor markers might be a result of the severity of IPF rather than an indication of recurrence. To our knowledge, this is the only case report demonstrating complete normalization of the three serum tumor markers—CEA, CA15‐3, and CA27‐29—following a single lung transplant. Increasing awareness of this phenomenon in patients with IPF, who are candidates for lung transplantation, could help prevent unnecessary scans and tumor marker tests, thereby avoiding potential delays in accessing this lifesaving procedure.

**Abbreviations**
ASCO: American society of clinical oncology
ATS: American thoracic society
BC: breast cancer
CEA: carcinoembryonic antigen
COPD: chronic obstructive pulmonary disease
ER: estrogen receptor
HER2: human epidermal growth factor receptor 2
IPF: idiopathic pulmonary fibrosis
MBC: metastatic breast cancer
NCCN: national comprehensive cancer network
PET‐CT: positron emission tomography‐computed tomography
PR: progesterone receptor

## 1 Introduction

Breast cancer (BC) is the most common cancer affecting women in the US and the second leading cause of cancer related mortality in women after lung cancer [1]. Tumor markers can be found in patients' body tissue, blood, or urine and can be elevated due to the presence of cancer [2]. Tumor markers can provide additional information about a patient's prognosis and response to treatment and can potentially detect disease response to treatment or progression before symptomatic or imaging detection. However, serum tumor markers are not well established in BC management and differing immunoassays and cutoffs impede their standardization and clinical utilization [3, 4]. Carcinoembryonic antigen (CEA), CA15‐3, and CA27‐29 are the three tumor markers recommended by the National Comprehensive Cancer Network (NCCN) to monitor disease activity in metastatic BC (MBC) [5]. All three biomarkers have prognostic significance in patients with MBC and are valuable in monitoring for tumor progression and response to therapy [4, 5]. Conversely, they have poor sensitivity and specificity in early‐stage BC and there is a lack of prospective randomized control trials to establish their use in these patients [3, 4].

CEA is a tumor marker frequently used for several types of cancer such as breast, colorectal, gastrointestinal, ovarian, uterine, and lung cancers [2, 3]. Nevertheless, it can have a high rate of false positivity and lack sensitivity since it could also be elevated due to non‐cancerous conditions like inflammatory bowel disease, cigarette smoking, and peptic ulcers [3]. Likewise, CA15‐3 has high false positivity and low specificity for BC since it could also be elevated due to benign breast disease or benign liver disease [2, 3]. Similarly, CA27‐29 can be found elevated for various reasons, such as in patients with benign conditions like ovarian cysts or several types of malignancies (lung, liver, and colon cancers) [2, 3]. Hence, guidelines established by the NCCN and the American Society of Clinical Oncology (ASCO) recommend their use in patients with MBC but not as surveillance in patients with early‐stage BC [3, 4, 5, 6].

The most common and progressive form of pulmonary fibrosis is idiopathic pulmonary fibrosis (IPF) [7, 8]. IPF is a chronic fibrotic lung disease characterized by dyspnea, fatigue, cough, compromised gas exchange, respiratory failure, and eventually death within 3–5 years of diagnosis [9]. The American Thoracic Society (ATS) has guidelines on how to diagnose a patient with IPF. This is done through a combination of clinical evaluation such as fine crackles at the base of the lung, pulmonary function testing, imaging, and sometimes biopsy. These assessments help diagnose IPF and exclude other known causes of interstitial lung disease (ILD) such as environmental exposures, medications, or connective tissue disease [10]. However, the hallmark criteria for IPF diagnosis are having radiological features as seen in high‐resolution computed tomography (CT) that demonstrate an interstitial pneumonia pattern characterized by honeycombing, basal and subpleural predominance, and reticular abnormalities [10]. Final diagnosis is confirmed through a multidisciplinary discussion typically amongst a pulmonologist and a radiologist [10]. A pathologist would be involved if a biopsy was performed, although a surgical lung biopsy is only necessary if the CT is indeterminate [10].

Most patients with IPF will require a lung transplant to survive since a transplant may be the only life‐extending option left for them [8, 11]. However, candidates are carefully screened and undergo rigorous evaluation to maximize post‐transplant survival benefit due to the prevailing organ shortage [12]. It is not advisable to offer a lung transplant to patients with a high risk for malignancy recurrence since this is an absolute contraindication. At least a 5‐year disease‐free interval should be demonstrated in patients with a history of BC, and some patients may have a recurrence risk that remains too high for lung transplant even after the 5‐year disease‐free interval [11, 12]. Moreover, some lung transplant centers may use tumor markers as a screening tool in pretransplant workup to potentially detect occult existing malignancy [13, 14]. Nevertheless, this is not advisable as tumor markers such as CEA, CA15‐3, and CA27‐29 may be high due to end‐stage lung disease and reflect an underlying lung inflammatory process instead of an actual malignancy [13, 14, 15, 16, 17].

Herein we present a case of a patient who had early‐stage BC, and after she completed her adjuvant chemotherapy, she developed IPF and persistent elevation of her CEA, CA15‐3, and CA27‐29 levels. Interestingly, to our knowledge, this is the first case report where all three tumor markers decreased to a normal range after her successful single lung transplant.

## 2 Case Presentation

**FIGURE 1:**
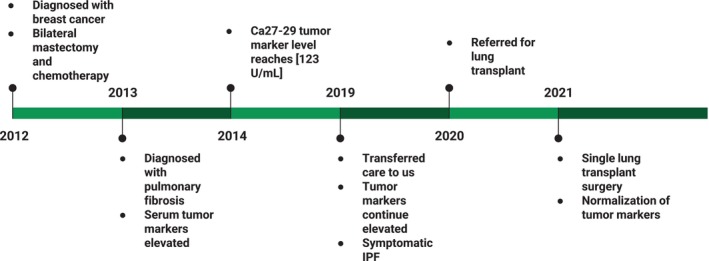
Timeline of main event from BC diagnosis to normalization of tumor markers.

A 52‐year‐old Hispanic woman with a family history of a brother dying from pulmonary fibrosis developed early‐stage BC. She was diagnosed at Hackensack University Medical Center with a left stage IIB (T2N1), estrogen receptor (ER) positive, progesterone receptor (PR) positive, and human epidermal growth factor receptor 2 (HER2) negative, Nottingham grade 2 invasive ductal carcinoma in 2012 (Figure 1). She opted for a bilateral mastectomy and left axillary lymph node dissection. Final pathology showed a 2.2 cm tumor and one out of 18 lymph nodes removed was positive for carcinoma. The margins of resection were free of tumor. She received adjuvant chemotherapy with dose dense adriamycin in combination with cyclophosphamide followed by paclitaxel. No adjuvant radiation was given. She then started adjuvant endocrine therapy. She took tamoxifen and different aromatase inhibitors on and off due to poor tolerance. Most recently she was on anastrozole. She completed almost 10 years of adjuvant endocrine therapy.

In 2013, after completing chemotherapy she was diagnosed with pulmonary fibrosis, which progressively worsened eventually leading to oxygen dependency. Her treating physician at that time ordered serum tumor markers and they were elevated. For this reason, she had a positron emission tomography‐computed tomography (PET‐CT) scan which was negative for recurrence. In 2019, she transferred care to us and due to her continued elevated tumor markers, we continued to monitor them periodically around every 6 months. They remained elevated from 2013 to 2021. Due to this elevation, the patient has had multiple scans showing no evidence of recurrence.

**FIGURE 2:**
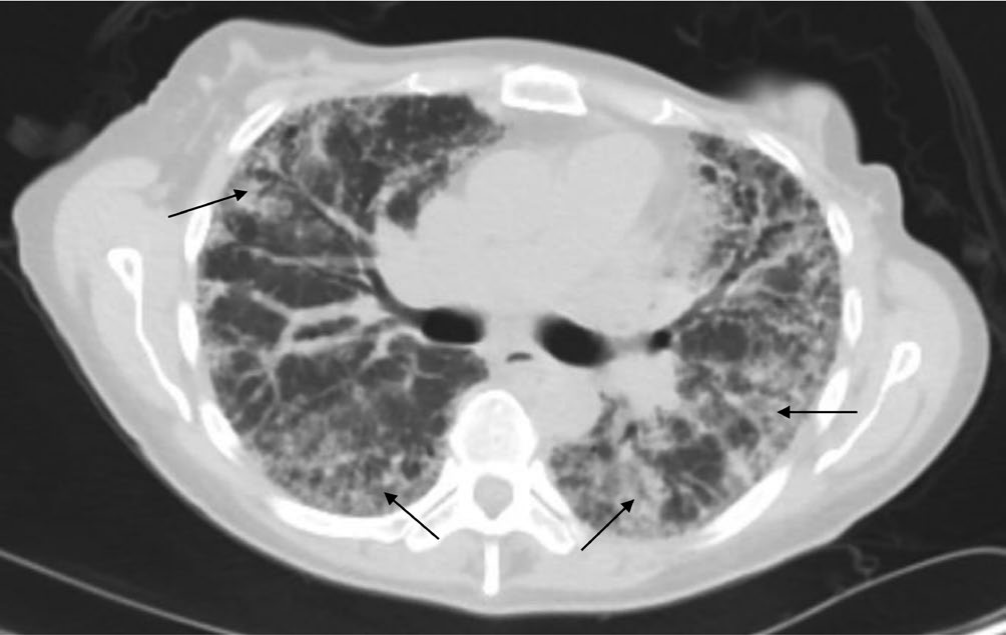
High resolution computed tomography of the chest showing end‐stage extensive pulmonary fibrosis. Trans axial image demonstrates areas of interstitial fibrosis with predominant involvement of periphery and lung bases. Arrow indicates areas of fibrotic regions.

In 2020, 8 years after her initial BC diagnosis, she was referred for lung transplant due to end‐stage lung disease resulting from IPF. The diagnosis of IPF in the patient was established following ATS clinical practice guidelines. Her high‐resolution CT scan of the chest showed end‐stage extensive pulmonary fibrosis (Figure 2). She had interstitial fibrosis with predominant involvement of the periphery, lung bases associated with traction bronchiectasis, and honeycombing. Moreover, there were irregular peripheral areas of parenchymal opacity potentially representing areas of bronchiolitis obliterans. Her IPF diagnosis was confirmed through a multidisciplinary discussion involving the pulmonologist, radiologist, and the rest of her medical team. After going through other hospitals, she was evaluated and enrolled through the Jackson health system at its Miami transplant institute. Due to the scarcity of organ procurement, she was given the possibility for a single lung transplant as a life‐saving option. During her evaluation, her physical exam was positive for tachypnea, clubbing in bilateral hands, decreased breath sounds, and rales bilaterally. Her breast exam showed bilateral reconstructed breasts with implants, no masses, and no lymphadenopathy. Her serum CEA, CA15‐3, and CA27‐29 continued to be elevated. She had a PET‐CT that was negative for metastasis. Mammography was not recommended because she had bilateral mastectomies. Sputum cytology was negative.

**FIGURE 3:**
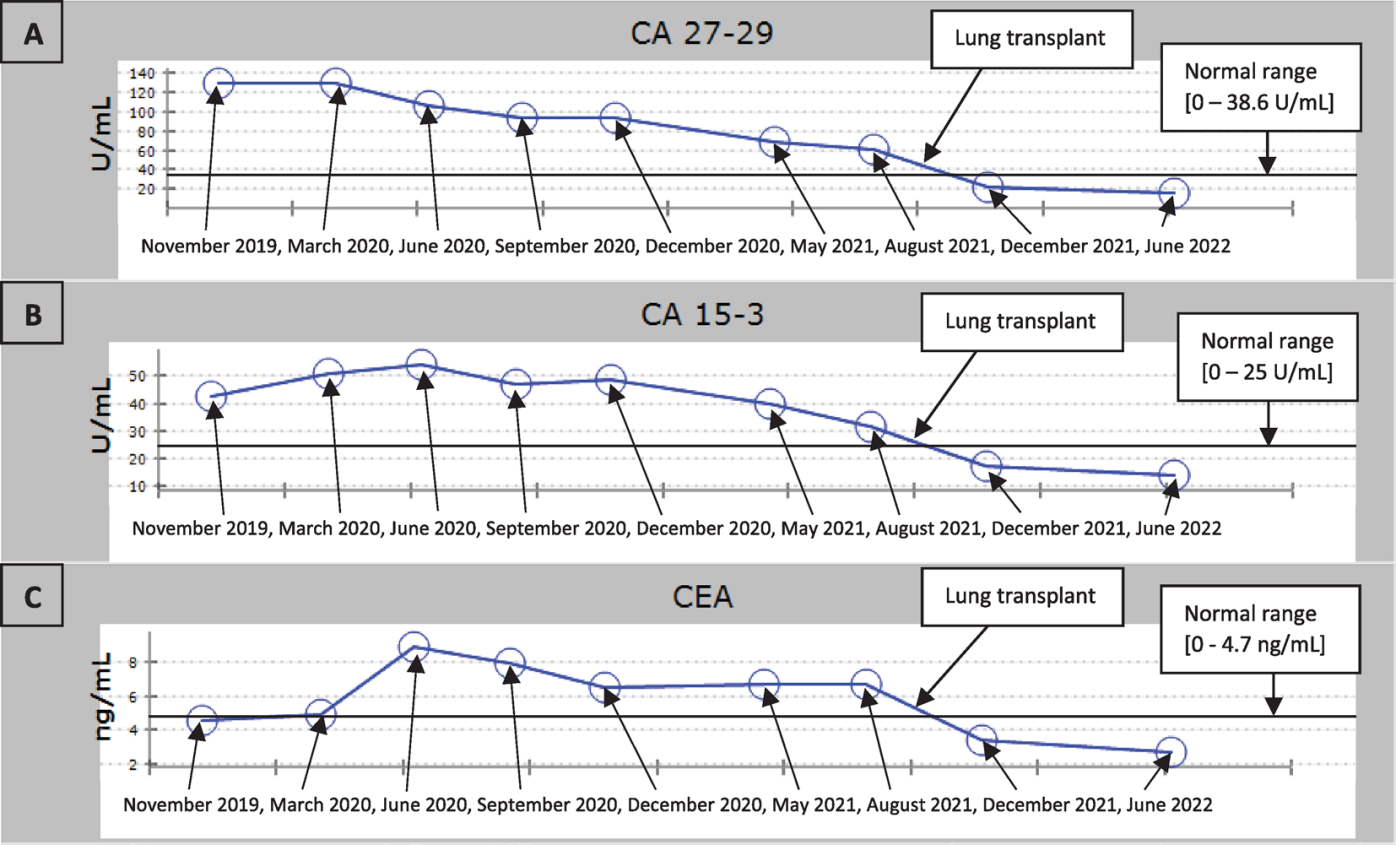
Tumor marker levels before and after lung transplant. (A) CA27‐29 tumor marker levels; (B) CA15‐3 tumor marker levels; (C) CEA tumor marker levels.

She was able to successfully undergo the procedure and received a left lung transplant in October of 2021. This lung transplant surgery was performed 9 years after being diagnosed with BC and 8 years after being diagnosed with interstitial pulmonary fibrosis. She had induction with basiliximab and started immunosuppression with prednisone, mycophenolate mofetil, and tacrolimus. The American College of Chest Physicians state that these immunosuppressants do not alter CEA, CA15‐3, and CA27‐29 tumor markers, including in patients that have lung disease without malignancy [18]. After her lung transplant, repeat tumor markers immediately started coming down and returned to normal ranges (Figure 3).

**FIGURE 4:**
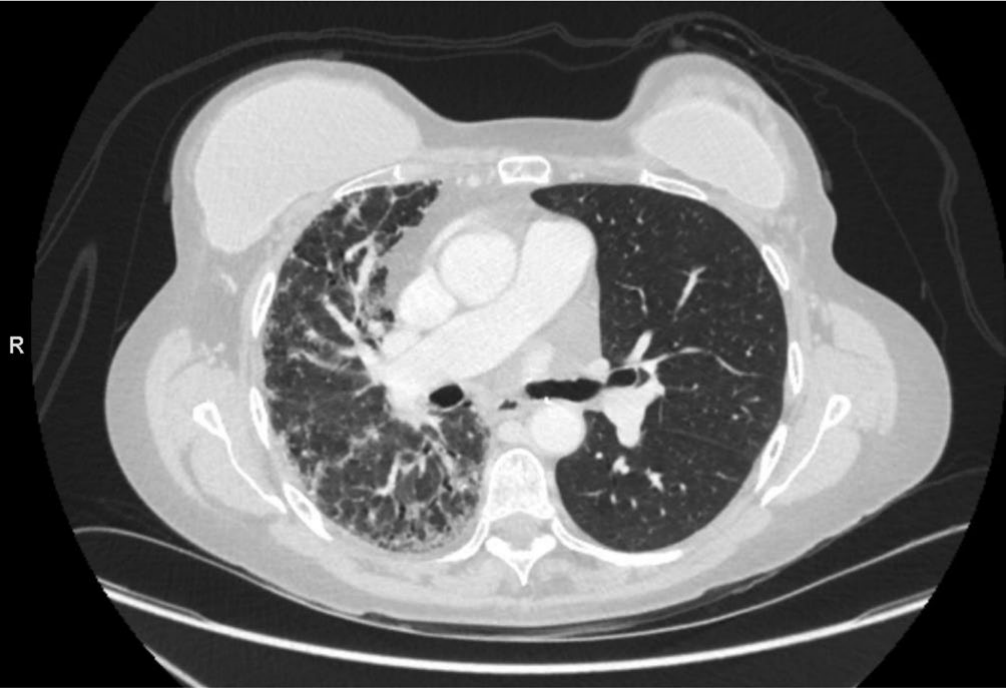
Axial contrast‐enhanced computed tomography scan of the chest demonstrating asymmetric lung findings. The right lung demonstrates diffuse interlobular septal thickening with areas of cylindrical bronchiectasis and moderate associated mosaic ground‐glass attenuation as well as honeycombing. Patient is status post left‐sided lung transplant.

It has now been years since her lung transplant and all her follow‐up tumor markers continue to be within normal ranges. The three markers assessed with their normal ranges are as follows: CEA with a normal range of [0–4.7 ng/mL], CA15‐3 with a normal range of [0–25 U/mL], and CA27‐29 with a normal range of [0–38.6 U/mL]. Her last follow‐up scans at our institution were in January 2025. Follow‐up chest CT, abdominal CT, and breast MRI showed that there has been no evidence of breast cancer recurrence. Chest CT demonstrated asymmetrical lung findings as expected between her right lung and her left transplanted lung (Figure 4). She is now happy living a healthy, active life alongside her family.

## 3 Discussion

Serum biomarkers CEA, CA15‐3, and CA27‐29 can be used to help monitor disease progression in MBC [2, 3, 4]. ASCO and NCCN guidelines recommend serial monitoring of these tumor markers in the metastatic setting, and an elevation of 20% to 30% in them, along with clinical examination, could indicate that treatment is failing [4]. However, use of tumor markers is limited to the metastatic setting and should not be used in patients with early‐stage BC. Oncologist nor lung transplant centers should use these tumor markers as a screening tool for occult malignancy because they demonstrate poor sensitivity and specificity. Monitoring tumor markers in early‐stage BC is not recommended because of their lack of specificity that can result in additional unnecessary tests and anxiety for the patient if they are elevated [5]. These tumor markers could be elevated due to other cancers or for non‐cancerous conditions as in the case of our patient.

The current surveillance guidelines for breast cancer recommend performing a history and physical exam 1–4 times a year for the first 5 years and annually thereafter [5]. Breast imaging is not indicated in patients who had mastectomies and reconstruction [5]. NCCN guidelines state that there is no indication for laboratory or imaging studies for metastasis screening in the absence of clinical signs and symptoms suggestive of recurrent disease [5]. The use of other biomarkers, such as circulating tumor cells (CTCs) or circulating tumor DNA (ctDNA), is currently under investigation; however, these other biomarkers are promising because they have more sensitivity and specificity [4, 19, 20]. While there are approved commercially available tests using these other biomarkers, these tests and biomarkers have not yet been endorsed by the NCCN and ASCO guidelines [4, 19]. In the future, the use of liquid biopsy and specialized biomarkers may lead to more personalized medicine and could be potentially integrated into the standard of care. However, there are currently no clinical practice guidelines that recommend their routine use in early‐stage BC surveillance [21].

In patients with lung disease, tumor markers can be abnormal. For example, in patients with idiopathic pulmonary fibrosis, tumor markers may be elevated due to the severity of the disease and not due to malignancy [16, 17]. A previous case report showed a patient with BC and IPF who had elevated tumor marker CA27‐29 and after bilateral lung transplant surgery, her tumor marker level returned to a normal range [22]. There is also evidence from a case series report in which previously treated BC patients with pulmonary fibrosis had persistent elevated CA27‐29 despite no evidence of malignant disease [15]. A study by Rusanov et al. showed that elevated tumor marker levels decreased in patients with IPF following lung transplant, with CA15‐3 having the greatest decrease [17]. In contrast, patients with chronic obstructive pulmonary disease (COPD) who had elevated tumor markers typically exhibited lower levels compared to those with IPF, and their markers did not necessarily decrease after lung transplant [17]. When studying the immunohistochemical tissue postmortem of lungs from patients with IPF, CEA staining was evident in the epithelia of respiratory bronchioles and alveoli [17, 23]. It was particularly increased in the alveoli region where type 2 pneumocytes proliferate [17, 23]. This information could be useful in assessing the degree of pathological changes due to IPF [17, 23]. Previous researchers have postulated that the high tumor marker levels seen in native lungs of patients are associated with the severity of the fibrotic tissue [14, 16, 17, 23].

Even though CEA is used as a screening tool for occult malignancy in several lung transplant centers, research has found that CEA levels in patients who were found to have lung cancer were similar to those of lung transplant patients who did not have lung cancer [17]. In addition, Okabe et al. performed a retrospective chart review which analyzed the survival rate of patients with high CEA levels and patients with normal CEA levels who underwent a lung transplant [14]. He found that the 5‐year survival rates of patients with high CEA levels was 84.0% and patients with normal CEA levels was 88.2%, which was not statistically significant [14]. This indicated that CEA levels were not a good predictor of posttransplant survival [14]. Furthermore, patients with IPF have higher pretransplant CEA levels compared with other end‐stage lung disease patients [13]. High serum CEA levels seem to indicate an inflammatory process in the native lung of patients [14, 16]. For these reasons, the CEA levels should not be used to make decisions for transplant eligibility.

Similarly, elevated CA15‐3 levels are not specific for malignancy in patients with pulmonary disease but may indicate lung epithelial injury and fibrosis [24, 25]. High CA15‐3 is commonly associated with interstitial lung disease, pulmonary fibrosis, and other forms of lung injury that reflect epithelial cell damage and increased mucin 1 (MUC1) release rather than being indicative of a malignancy [24, 25]. Furthermore, increased CA15‐3 levels could be associated with pathological leakage of the epithelial cells into the bloodstream and a decreased CA15‐3 clearance rate [24]. In Rusanov et al. study, CA15‐3 was specially elevated in patients with IPF and their levels decreased after lung transplant [17]. Likewise, CA27‐29 can be elevated in various non‐malignant lung disorders like IPF and other interstitial lung disease due to epithelial inflammation and injury related to MUC1 shedding similarly to CA15‐3 [15, 22].

Based on the foregoing, tumor marker elevation in the context of fibrotic lung disease remains a diagnostic challenge. In our patient, evaluating several tumor markers led to increased anxiety and extensive unnecessary work‐up due to their elevation for many years. Moreover, there is limited information showcasing how IPF alters tumor markers CEA, CA15‐3, and CA27‐29. Our manuscript adds knowledge to this area because our patient had these three elevated tumor markers that all normalized after a single lung transplant without the need of a double lung transplant. The normalization of markers after the single lung transplant supports the premise that the elevation was likely secondary to pathological changes from IPF. To our knowledge, this is the only case report that shows complete reversal of all three serum tumor markers CEA, CA15‐3, and CA27‐29 after a single lung transplant.

## 4 Conclusion

Since it is known that all three tumor markers CEA, CA15‐3, and CA27‐29 lack specificity and sensitivity in early‐stage BC, they should not be used to monitor cancer recurrence. Their elevation can lead to increased stress and unnecessary tests.

Although there is not much evidence about these three tumor markers directly decreasing after lung transplant, which is what makes our case report unique, there is evidence that these tumor markers can be elevated in patients with inflammatory lung conditions. By increasing awareness of the possibility of pulmonary fibrosis potentially causing elevated CEA, CA15‐3, and CA27‐29, future lung transplant patients should not have to undergo unnecessary serum tumor marker tests to be eligible for a potentially lifesaving lung transplant. Newer biomarkers are currently in development, such as circulating tumor DNA. In the near future, we might be able to use more specific biomarkers in early‐stage BC to monitor for recurrence and even guide therapies.

## Author Contributions

Y.C. and A.C.S.‐L. contributed to the concept and design of the manuscript. Y.C. and A.C.S.‐L. performed the search and writing of the manuscript. Y.C., C.L.V., and A.C.S.‐L. contributed to editing and revising the manuscript. All authors read and approved the final manuscript.

## Funding

The authors have nothing to report.

## Ethics Statement

The authors have nothing to report.

## Consent

Written informed consent was obtained from the patient for publication of this case report and any accompanying images. A copy of the written consent is available for review by the Editor‐in Chief of this journal.

## Conflicts of Interest

C.L.V. declares consulting/advisory relationship and honoraria with Veru, Biotheranostics, Novartis, and Total Health Conferencing. A.C.S.‐L. declares consulting/advisory relationship with Merck, Sanofi/Aventis, Oncocyte, AstraZeneca, Gilead Sciences, Stemline Therapeutics, Guardant Health, Sermonix; research funding from AstraZeneca, Seagen, OBI Pharma. Y.C. declare no conflicts of interest. The authors' consulting/advisory relationships and receipt of honoraria from pharmaceutical companies had no involvement in this case report.

## Data Availability

The authors have nothing to report.
